# Supplementary material for: Building a community-based open harmonised reference data repository for global crop mapping
Source: PLoS One. 2023 Jul 13;18(7):e0287731. doi: 10.1371/journal.pone.0287731 (PMC10343028; doi:10.1371/journal.pone.0287731)
Supplement: S1 Table — (DOCX) [file pone.0287731.s001.docx]

**Table S1** WorldCereal harmonization protocol (see Zenodo 10.5281/zenodo.7584463 for supporting files)

| 1 | Check the selection criteria on available geo-location, timestamp, and recent years (>=2017). A data set should qualify these criteria. |
| --- | --- |
| 2 | Prepare data in the desired format: geopackage or shapefile in case of point and polygon geometry and geotiff in case of rasters. Co-ordinate system must be in WGS84. Finally, a logical filename must be defined. See WorldCereal_FileFormat.xlsx for detailed filename convention. |
| 3 | Add minimum required attributes which are:   - sampleID (unique ID) - valtime (indicating for which date this observation is valid) - LC (land cover code, see WorldCereal legends) - CT (crop type code, see WorldCereal legends) - IRR (irrigation code, see WorldCereal legends)   See WorldCereal_FileFormat.xlsx for the attribute conventions. |
| 4 | Assess a proper validity date. The WorldCereal system needs an observation date. If you do not have a real date you need to derive the date from the year (and season) of observation. This is further explained in the document DerivingValidityTime.pdf. |
| 5 | Map to the WorldCereal legends (LC, CT, IRR). The legends are defined in the document WorldCereal_LC_CT_IRR_legends.xlsx. Note that CT has 4 levels. In general, it is advised to map to the most detailed CT-level to preserve maximum information. |
| 6 | Define limited meta data for personal use such as a title, data type (FieldObservationSurvey, ClassificationValidatedCrowd/Expert, FormalDeclaration, AutomatedClassification) and a personal assessment of the data confidence on a range of 50 (poor) to 100 (high). If an owner would like to share a data set for public use, complete metadata must be added (see MetaDataTemplate.xlsx). One of the metadata elements is the confidence score. To value the confidence score, a set of rules has been developed to assess the spatial, temporal, and thematic accuracy (see ReferenceDatasetsConfidenceScoreCalculations.pdf and DataQuality_Calculator.xlsx). In case of spatial accuracy separate instructions are available (see Protocol to assess spatial accuracy.pdf). |
