# Supplementary material for: Building a community-based open harmonised reference data repository for global crop mapping
Source: PLoS One. 2023 Jul 13;18(7):e0287731. doi: 10.1371/journal.pone.0287731 (PMC10343028; doi:10.1371/journal.pone.0287731)
Supplement: S2 Table — (DOCX) [file pone.0287731.s002.docx]

**Table S2** Overview of reference data leads collected in WorldCereal (FO = Field Observation; CV = Classification or Validation by crowd or expert; FD = Formal Declaration; AC = Automated Classification)

| Name data lead | Description | Data type | Reference |
| --- | --- | --- | --- |
| 50by2030 | The 50x2030 Initiative supports low- and lower-middle income countries in gathering foundational agricultural and rural data from 2019 to 2030. So far, some maps are available:  1) https://datacatalog.worldbank.org/dataset/high-resolution-crop-and-maize-area-mapping-ethiopia  2) https://datacatalog.worldbank.org/dataset/high-resolution-crop-and-maize-area-mapping-malawi  In-situ data might come available later. | AC, (FO) | www.50x2030.org |
| AAFC Crop Inventory | The Earth Observation Team of the Science and Technology Branch (STB) at Agriculture and Agri-Food Canada (AAFC) collected ground truth data via windshield surveys. | FO | https://open.canada.ca/data/en/dataset/503a3113-e435-49f4-850c-d70056788632 |
| BAGE | Buenos Aires Grain Exchange (BAGE) is a non-profit organization, a typical intermediate service provider. BAGE organizes crop tours in Argentina during which ground data are collected like crop type from plots at both sides of a location. | FO | www.bolsadecereales.com |
| CAWa project | A crop type dataset for consistent land cover classification in Central Asia, 2018. | FO | doi.org/10.1038/s41597-020-00591-2 |
| CGIAR-CIMMYT | CGIAR (formerly the Consultative Group for International Agricultural Research) is a global partnership that unites international organizations engaged in research about food security. For example, the International Maize and Wheat Improvement Center (CIMMYT) and their disease monitoring network in eastern and southern Africa. | FO | [www.cgiar.org](http://www.cgiar.org) |
| CGIAR-GARDIAN | GARDIAN, the Global Agricultural Research Data Innovation & Acceleration Network, is the CGIAR flagship data harvester. GARDIAN enables the discovery of publications and datasets from the thirty-odd institutional publications and data repositories across all CGIAR Centers. | FO | gardian.bigdata.cgiar.org |
| CONAB | Companhia Nacional de Abastecimento (CONAB) is a public company based in Brasilia, linked to the Ministry of Agriculture, Livestock and Supply (MAPA). CONAB has field data and maps of soybeans and maize (summer crops) and irrigated rice of Brazil. | FO, AC | www.conab.gov.br |
| COPERNICUS-GEOGLAM | Data are produced by the Copernicus4GEOGLAM service of Copernicus Global Land in various countries. Data includes (among others): i) ground reference data collected during field survey (georeferenced points and polygons, photos) and ii) in-season and end-of-season crop type maps and crop masks; iii) crop area estimates. | FO | land.copernicus.eu/global/about-copernicus4geoglam; https://data.jrc.ec.europa.eu/collection/id-00356 |
| Copernicus hotspot LCCE | Copernicus HotSpot Land Cover Change Explorer. | CV | land.copernicus.eu/global/hsm |
| Croplands.org | Croplands.org is part of the Global Food Security Analysis-Support Data at 30 Meters (GFSAD30) project. Global land-use data are collected with a mobile application. | FO | croplands.org |
| Digital Earth Africa | DEA produces crop type maps of Africa. Label data is based on interpretation of high-resolution imagery and other reference data. They also offer cropland extent maps. | CV,AC | www.digitalearthafrica.org |
| ESA projects | Relevant ESA (European Space Agency) projects such as Sen2Agri and Sen4Stat mainly focusing on Africa. | FO | [esa-sen2agri.org](http://www.esa-sen2agri.org),  [esa-sen4stat.org](http://www.esa-sen4stat.org) |
| ESYRCE-Spain | Encuesta sobre Superficies y Rendimientos Cultivos (ESYRCE) - Crop surfaces and crop yield survey  Classification by Ministry of Agriculture, Fisheries and Food of Spain | FO | https://www.mapa.gob.es/es/ |
| EUROCROPS | EuroCrops is a dataset for automatic vegetation classification from multi-spectral and multi-temporal satellite data, annotated with official LPIS (Land Parcel Identification System) reporting data from countries of the European Union, harmonized by the Technical University of Munich and GAF AG. | FD | www.eurocrops.tum.de |
| Eurostat LUCAS | The Land Use/Cover Area frame Survey (LUCAS) is a harmonised in situ land cover and land use data collection exercise that extends over the whole of the EU’s territory. An in-situ survey implies that data are gathered through direct observations made by surveyors on the ground. It is based on a standardised survey methodology in terms of a sampling plan, classifications, and data collection processes. | FO | ec.europa.eu/eurostat/web/lucas |
| FAO-EOSTAT / AGRIS | In-situ data collected in Senegal, Afghanistan, Uganda, and Lesotho by national agencies. | FO | www.50x2030.org |
| FAO-WAPOR | Field data collected in the frame of FAO Water Productivity Open-access portal (WaPOR). | FO | https://www.fao.org/in-action/remote-sensing-for-water-productivity/en/ |
| IIASA | IIASA collects reference data via tools such as Geo-Wiki and LACO-Wiki as well as mobile apps such as PicturePile and CropObserve. | CV, FO | [www.geo-wiki.org](http://www.geo-wiki.org), [www.laco-wiki.net](http://www.laco-wiki.net) |
| INPE-LEM | LEM+ dataset (Luís Eduardo Magalhães) was developed the Brazilian Instituto Nacional de Pesquisas Espaciais (INPE). | FO | www3.inpe.br, doi.org/10.17632/vz6d7tw87f.1 |
| INTA-field data | In situ data collected by INTA (Instituto Nacional de Tecnología Agropecuaria) in the Buenos Aires province during the growing season 2018-2019. INTA also has HR crop type maps for different regions in Argentina. | FO, AC | - |
| JECAM sites | The Joint Experiment for Crop Assessment and Monitoring (JECAM) is an initiative created by the GEO Agriculture Monitoring Community of Practice. | FO | jecam.org |
| JECAM-CIRAD | Harmonized in situ JECAM datasets for agricultural land use mapping and monitoring in tropical countries. | FO | www.cirad.fr/en,  10.18167/DVN1/P7OLAP |
| LISTA-field-data | In-situ data collected by LISTA (Laboratorio de Investigación y Servicios en Teledeteccion de Azul), Argentina. | FO | - |
| LPIS | European land-parcel identification systems (LPIS) of Belgium (Flanders), Latvia, France, and Austria. | FD | Austria: www.data.gv.at  Belgium: https://lv.vlaanderen.be/en  France: www.ign.fr/institut  Latvia: www.lad.gov.lv/en |
| LUCAS 2018 Copernicus | Through geo-spatial analysis and by semantically linking the LUCAS core and Copernicus land cover observations, 58,428 polygons are provided with a level-3 land cover (66 specific classes including crop type) and land use (38 classes) information as inherited from the LUCAS core observation. | FO | ec.europa.eu/jrc/en,  doi.org/10.6084/m9.figshare.12382667.v3 |
| N2Africa | Field survey data collected in the frame of the N2Africa project which aims to improve the legume cropping systems. | FO | www.n2africa.org |
| NASA Harvest | Consortium of multidisciplinary and multisectoral actors from around the world, led and implemented by the Harvest Hub at the University of Maryland.  NASA Harvest recently published the CropHarvest dataset, a crop dataset of geo-referenced labels with satellite data inputs, each consisting of latitude, longitude, the associated crop type label, and a satellite pixel time series. | FO | nasaharvest.org,  github.com/nasaharvest/cropharvest |
| Radiant MLHub | Non-profit organization actively working to develop Earth observation machine learning libraries and models through an open source hub. Fosters a community of practice to develop standards around machine learning for Earth observation. Offers access to several, well annotated in-situ data sets via ML hub. | FO | [www.radiant.earth](http://www.radiant.earth), mlhub.earth |
| OneAcreFund-MEL | Data from Monitoring, Evaluation and Learning (MEL) agronomic surveys in Kenya, Rwanda and Tanzania, 2016-2019 published by OneAcrefund. | FO | oneacrefund.org |
| OSF-AfSIS | GeoSurvey and MobileSurvey based data on Land Cover Classification (LCC), crop species, crop type and livestock occurrence predictions for Tanzania. | FO | osf.io/4ngau |
| SIGPAC | Agricultural Plot Geographic Information System (SIGPAC) is a geographic information system and reference system for the identification of agricultural parcels. Used for management and control of the aid regimes established by the regional authorities of Spain. | FD | Catalunya: [agricultura.gencat.cat](http://agricultura.gencat.cat)  Andalucia: www.juntadeandalucia.es |
| Soils4Africa | (Planned) field surveys on soil and land cover. | FO | www.soils4africa-h2020.eu |
| WFP-field-survey | World Food Program (WFP) crop field surveys | FO | www.wfp.org |
| Maps | | | |
| Agrimetrics Field Explorer | Commercial app giving access to 2.8 million UK fields. They use crop data from CEH Land Cover Plus Crop map (2015-19). See below. | AC | agrimetrics.co.uk/products/field-explorer |
| CEH Land Cover Plus Crop map (2015-19) | UKCEH Land Cover® Plus Crop maps: digital maps of cropping in Great Britain. Two million land parcels are categorised within the Land Cover Map spatial framework, providing information on annual crop types for every field in Great Britain. | AC | www.ceh.ac.uk/crops2015#obtaining |
| CROME | The Crop Map of England (CROME) is a polygon vector (hexagonal polygons rather than field boundary polygons) dataset mainly containing the crop types and land cover of England. The classification was created automatically using supervised classification (Random Forest Classification) from the combination of Sentinel-1 Radar and Sentinel-2 Optical Satellite images. Available for 2017-2019. | AC | data.gov.uk/dataset/fb19d34f-59e6-48e7-820a-fe5fda3019e5/crop-map-of-england-crome-2018 |
| Cropland Data Layer (CDL) - USDA | The CDL is a raster, geo-referenced, crop-specific (plus cultivated) land cover data layer created annually for the continental United States using moderate resolution satellite imagery and extensive agricultural ground truth. | AC | www.nass.usda.gov/Research_and_Science/Cropland/Release,  nassgeodata.gmu.edu/CropScape |
| FROM-GLC10 | FROM-GLC (Finer Resolution Observation and Monitoring of Global Land Cover) is a 30 m resolution global land cover maps produced using Landsat Thematic Mapper (TM) and Enhanced Thematic Mapper Plus (ETM+) data. The 10 m version was released in March 2019. | AC | data.ess.tsinghua.edu.cn,  data.ess.tsinghua.edu.cn/fromglc2017v1.html |
| GLAD-UMD | Annual soybean maps for south America at 30m resolution for 2000-2019.  Global land cover and land use 2019, v1.0. | AC | glad.umd.edu/projects/commodity-crop-mapping-and-monitoring-south-america,  glad.umd.edu/dataset/global-land-cover-land-use-v1 |
| GlobeLand30 | Comprise ten types of land cover, including forests, artificial surfaces and wetlands, crop land, for 2000, 2010 and 2020. They were extracted from more than 20,000 Landsat and Chinese HJ-1 satellite images. | AC | www.globeland30.org |
| Map Navarre Spain | Crop Classification Based on Temporal Signatures of Sentinel-1 Observations over Navarre Province, Spain. | AC | www.mdpi.com/2072-4292/12/2/278 |
| Map north-east China | The 10-m crop type maps in Northeast China during 2017–2019. | AC | www.nature.com/articles/s41597-021-00827-9 |
| NASU-SSAU | Crop type map 10 m 2020. | AC | - |
