# Supplementary material for: Building a community-based open harmonised reference data repository for global crop mapping
Source: PLoS One. 2023 Jul 13;18(7):e0287731. doi: 10.1371/journal.pone.0287731 (PMC10343028; doi:10.1371/journal.pone.0287731)
Supplement: S3 Table — (DOCX) [file pone.0287731.s003.docx]

**Table S3** Citations and data license of original public data sets harmonized in WorldCereal

| **Dataset** | **Citation** | **Data license** |
| --- | --- | --- |
| AAFC Crop Inventory | Agriculture and Agri-Food Canada, 2020, “Annual Crop Inventory Ground Truth Data, 2011-2020”, Agroclimate, Geomatics and Earth Observation Division, Science and Technology Branch. | Open Government License – Canada (~CC_BY) (https://open.canada.ca/en/open-government-licence-canada) |
| CAWa project | Remelgado, R., Zaitov, S., Kenjabaev, S., Stulina, G., Sultanov, M., Ibrakhimov, M., Akhmedov, M., Dukhovny, V. and Conrad, C., 2020. A crop type dataset for consistent land cover classification in Central Asia. Scientific Data, 7(1), pp.1-6. doi.org/10.1038/s41597-020-00591-2 | CC_BY |
| CGIAR-GARDIAN | Fotso Kuate A, Hanna R, Doumtsop Fotio ARP, Fomumbod Abang A, Nanga Nanga S, Ngatat S, Tindo M, Masso C, Ndemah R, Suh C and KKM Fiaboe. 2019. Spodoptera frugiperda Smith (Lepidoptera: Noctuidae) in Cameroon: case study on its distribution, damage, pesticide use, genetic differentiation and host plants. Plos One. [doi.org/10.1371/journal.pone.0215749](https://doi.org/10.1371/journal.pone.0215749)  [doi.org/10.25502/40vn-jf91/d](https://doi.org/10.25502/40vn-jf91/d) | CC_BY |
|  | E. Jeroen Huising, Kamaludeen Tijjani, 2018, Results of the validation of OCP newly developed maize fertilizer formulations for the maize growing belt of Nigeria, Summary report, IITA. doi.org/10.25502/PAKR-Y904/D | CC_BY |
|  | International Food Policy Research Institute (IFPRI); Ghent University; University of Manchester. 2020. Crop Monitoring Using Smartphone Based Near-Surface Remote Sensing: Ground Pictures of Wheat and Auxiliary Data from Northern India. Washington, DC: IFPRI [dataset]. doi.org/10.7910/DVN/DBAFZY. Harvard Dataverse. Version 1. | CC_BY |
| COPERNICUS-GEOGLAM | European Commission, Joint Research Centre (JRC) (2021): Kenya AOI. European Commission, Joint Research Centre (JRC) [Dataset] PID: <http://data.europa.eu/89h/5b6245d3-e561-4f6c-8c09-627888063d11>  European Commission, Joint Research Centre (JRC) (2021): Tanzania AOI. European Commission, Joint Research Centre (JRC) [Dataset] PID: <http://data.europa.eu/89h/8f86f452-7a04-4f8f-bced-1796e23adc85>  European Commission, Joint Research Centre (JRC) (2021): Uganda AOI. European Commission, Joint Research Centre (JRC) [Dataset] PID: <http://data.europa.eu/89h/839bc122-205d-4644-9dd6-c37901ac0d63> | ~ CC_BY  (No limitations. Anybody can directly and anonymously access the data, without being required to register or authenticate. Reuse is authorised, provided the source is acknowledged (https://data.jrc.ec.europa.eu/licence/com_reuse) |
| CGIAR-CIMMYT | CIMMYT - disease monitoring Africa. The International Maize and Wheat Improvement Center (CIMMYT). https://www.cimmyt.org. | CC_BY_NC |
| Digital Earth Africa | Burton, C.; Halabisky, M.; Yuan, F.; Ongo, D.; Mar, F.; Addabor, V.; Mamane, B.; Adimou, S.; Chong, E.; Leith, A.; Lewis, A; Hall, L.; Jorand, C. Digital Earth Africa Cropland 2019 Reference Dataset, Version 1.0. | CC_BY  (https://docs.digitalearthafrica.org/en/latest/data_specs/Cropland_extent_specs.html#) |
| EUROCROPS | Schneider, Maja, & Körner, Marco. (2022). EuroCrops [Germany Lower Saxony]. Zenodo. doi.org/10.5281/zenodo.6937139 | CY_BY |
| FAO-WAPOR | FAO WaPOR, 2022. Field data collected in the frame of FAO Water Productivity Open-access portal (WaPOR). https://www.fao.org/in-action/remote-sensing-for-water-productivity/en/. | CY_BY  (confirmed by e-mail) |
| INPE-LEM | Oldoni, L. V.; Sanches, I. D.; Picoli, M. C. A.; Covre, R. M.; Fronza, J. G. 2020. LEM+ dataset: for agricultural remote sensing applications. Data in Brief (2020). doi.org/10.17632/vz6d7tw87f.1 | CY_BY |
| JECAM-CIRAD | Jolivot, Audrey; Lebourgeois, Valentine; Ameline, Mael; Andriamanga, Valerie; Bellon, Beatriz; Castets, Mathieu; Crespin-Boucaud, Arthur; Defourny, Pierre; Diaz, Santiana; Dieye, Mohamadou; Dupuy, Stephane; Ferraz, Rodrigo; Gaetano, Raffaele; Gely, Marie; Jahel, Camille; Lelong, Camille; Le Maire, Guerric; Leroux, Louise; Lo Seen, Danny; Muthoni, Martha; Ndao, Babacar; Newby, Terry; De Oliveira Santos, Cecilia Lira Melo; Rasoamalala, Eloise; Simoes, Margareth; Thiaw, Ibrahima; Timmermans, Alice; Tran, Annelise; Begue, Agnes, 2021, "Harmonized in situ JECAM datasets for agricultural land use mapping and monitoring in tropical countries", doi:10.18167/DVN1/P7OLAP, CIRAD Dataverse, V2 | CC_BY |
| LPIS-Latvia | LPIS Latvia, 2019. Rural Support Service Republic of Latvia. lad@lad.gov.lv. https://www.lad.gov.lv/en | CC-BY  ("We don’t have any publishing restriction about open data, which is available in the provided link" (Ministry of Agriculture, 2021); https://github.com/maja601/EuroCrops/wiki/Latvia) |
| LPIS-France | France, IGN Registre parcellaire graphique (RPG) 2016-2019 (https://www.data.gouv.fr/fr/datasets/registre-parcellaire-graphique-rpg-contours-des-parcelles-et-ilots-culturaux-et-leur-groupe-de-cultures-majoritaire/) | Open License (~CC_BY)  https://www.etalab.gouv.fr/wp-content/uploads/2014/05/Open_Licence.pdf |
| LPIS-Belgium | LPIS Belgium, Department of Agriculture and Fisheries of the Flemish government (Belgium) | Open License (~CC_BY)  https://overheid.vlaanderen.be/sites/default/files/documenten/ict-egov/licenties/hergebruik/modellicentie_gratis_hergebruik_v1_0.html |
| LPIS-Austria | LPIS Austria, © Agrarmarkt Austria als Geodatenstelle (https://www.data.gv.at/katalog/dataset/e21a731f-9e08-4dd3-b9e5-cd460438a5d9) | CC_BY |
| LUCAS 2018 Copernicus | LUCAS Copernicus 2018: Earth Observation relevant in-situ data on land cover throughout the European Union" by Raphaël d’Andrimont, Astrid Verhegghen, Michele Meroni, Guido Lemoine, Peter Strobl, Beatrice Eiselt, Momchil Yordanov, Laura Martinez-Sanchez and Marijn van der Velde. doi.org/10.5194/essd-13-1119-2021 | CC_BY |
| NASA Harvest - CropHarvest | Hannah Kerner, Gabriel Tseng, Inbal Becker-Reshef, Catherine Nakalembe, Brian Barker, Blake Munshell, 396 Madhava Paliyam, and Mehdi Hosseini. Rapid response crop maps in data sparse regions. In ACM 397 SIGKDD Conference on Data Mining and Knowledge Discovery Workshops, 2020 | CC_BY  (<https://github.com/nasaharvest/cropharvest/blob/main/datasets.md>) |
|  | CropHarvest: a global satellite dataset for crop type (Mali - polygon - multiple crop). NASA Harvest and Lutheran World Relief. <https://github.com/nasaharvest/cropharvest> | CC_BY  (<https://github.com/nasaharvest/cropharvest/blob/main/datasets.md>) |
|  | CropHarvest: a global satellite dataset for crop type (Zimbabwe - point - multiple crop). NASA Harvest and FEWS NET (Famine Early Warning Systems Network) and the Zimbabwe Ministry of Agriculture. https://github.com/nasaharvest/cropharvest | CC_BY_SA  (<https://github.com/nasaharvest/cropharvest/blob/main/datasets.md> |
| Radiant MLHub | Bocquet, C., & Dalberg Data Insights. (2019) "Dalberg Data Insights Uganda Crop Classification", Version 1.0, Radiant MLHub. Date Accessed: 8 December 2020. [doi.org/10.34911/RDNT.EII04X](https://doi.org/10.34911/RDNT.EII04X) | CC_BY_SA |
|  | Great African Food Company (2019) "Great African Food Company Tanzania Ground Reference Crop Type Dataset", Version 1.0, Radiant MLHub. Date Accessed: 8 December 2020. doi.org/10.34911/RDNT.5VX40R | CC_BY_SA |
|  | PlantVillage (2019) "PlantVillage Kenya Ground Reference Crop Type Dataset", Version 1.0, Radiant MLHub. Date Accessed: 7 December 2020. doi.org/10.34911/RDNT.U41J87 | CC_BY_SA |
| OneAcreFund-MEL | One Acre Fund (2020). Data from MEL agronomic surveys in Kenya, Rwanda and Tanzania, 2016-2019 | CC_BY |
| OSF-AfSIS | Walsh, Markus, Joel Meliyo, Bruce Scott, Barbara Walsh, and Bob Macmillan. 2021. “Tanzania Soil Information Service (TanSIS).” OSF. September 6. doi:10.17605/OSF.IO/4NGAU. | CC_BY |
| SIGPAC | SIGPAC Andalucia, @Junta de Andalucia | ~CC_BY  (<https://www.juntadeandalucia.es/organismos/agriculturaganaderiapescaydesarrollosostenible/areas/politica-agraria-comun/paginas/sigpac-descarga-informacion-geografica-shapes-provincias.html>) |
|  | SIGPAC Catalunya 2019. Departament d'Acció Climàtica, Alimentació i Agenda Rural. sigpac.daam@gencat.cat. https://agricultura.gencat.cat/ca/ambits/desenvolupament-rural/sigpac/descarregues/ | ~CC_BY  (In accordance with article 17.1 of Law 19/2014, the © Generalitat de Catalunya allows the reuse of the contents and data as long as the source and date of update are cited and the information is not distorted (Article 8 of Law 37/2007) and also that it does not contradict a specific license) |
| Cropland Data Layer (CDL) - USDA | USDA National Agricultural Statistics Service Cropland Data Layer 2019. Published crop-specific data layer [Online]. Available at https://nassgeodata.gmu.edu/CropScape/ (accessed 8 September 2020, verified 8 September 2020). USDA-NASS; Washington; DC. | CC_BY  (The NASS Cropland Data Layer has no copyright restrictions. The CDL is considered public domain and free to redistribute. However; NASS would appreciate acknowledgement for the usage of our CDL product.) |
