# Supplementary material for: Building a community-based open harmonised reference data repository for global crop mapping
Source: PLoS One. 2023 Jul 13;18(7):e0287731. doi: 10.1371/journal.pone.0287731 (PMC10343028; doi:10.1371/journal.pone.0287731)
Supplement: Attachment — Submitted filename: Response to Reviewers.docx [file pone.0287731.s004.docx]

Dear Reviewers,

We sincerely thank you for taking the time and effort for reviewing our manuscript “Building a community-based open harmonised reference data repository for global crop mapping”.

We heartily appreciated your comments that fundamentally confirmed the importance and relevance of this research. The table below summarizes our responses to each comment. We have revised our research paper in the light of these useful suggestions and comments and strongly believe that this revision has improved the paper.

Best regards,

Hendrik Boogaard

On behalf of all co-authors

Table: Responses to the reviewers

| **Issue** | **Response** | **Where?** |
| --- | --- | --- |
| **Reviewer 1** | | |
| 1 In Table 2, the collected data sets covered different years. The land cover types, particularly crop types usually change from year to year and sometimes the changes are considerable. If the datasets are used for classification, how to deal with the time/year difference? How to use these old samples for in-season classification for the current year? | The harmonized reference data set, described in this paper, served not only the WorldCereal project. The data can be used for all kind of mapping initiatives focussing on certain regions, crops and/or years. While for certain local applications the data set could be rather complete, this is not the case at global level. Global initiatives need to define approaches to deal with data gaps. Defining such approaches was not an objective of this paper. We refer to another WorldCereal paper (under review) to learn more about strategies to anticipate on the unequal distribution of reference data over years and space.  The discussion in the manuscript have been adapted to reflect this point.  Obviously, the harmonized data set described in this paper is just a first step. During our work we engaged with the GEOGLAM initiative (Group on Earth Observations Global Agricultural Monitoring Initiative), and we contributed to a new working group around in-situ data. In our paper we recommend an active role of GEOGLAM and the new working group, encouraging our community to publish reference data and to help filling gaps in time and space and to achieve a more balanced distribution over crop types.  *Van Tricht et al. WorldCereal: a dynamic open-source system for global-scale, seasonal, and reproducible crop and irrigation mapping. Earth System Science Data (ESSD), in review.* | Manuscript (line 283-284; line 294-296) |
| 2、 In Figure 3b, the percentage of other crop types is 60.5%. What are other crop types? The percentages of crop types are very imbalance. Will this affect the classification accuracy? | Ideally, the harmonized reference data set has a crop distribution reflecting the actual situation of the target region. Obviously, this is not the reality, and we must rely on the in-situ data that were shared within our community. This imbalance will influence the classification accuracy.  Assessing the effect of these imbalances on the classification accuracy was not the objective of this work. But we added this concern on crop distribution, affecting classification accuracy, to the discussion.  We also adjusted Figure 3b and we now show the data availability of the most relevant crop types. Besides wheat and maize, we added bars for rice, millet, sorghum, (sweet) potato and cassava. | Manuscript (line 295-297 and figure 3b) |
| 3、 Confidence score is very important for data fitness. Three individual scores on spatial, temporal, and thematic accuracy are averaged using specific weights. These weights allow to vary the importance of the three types of accuracy. How to calculate the weights of confidence score? | Indeed, assessing the data sets for a specific use is important. When we started our harmonization work, we could not find an existing approach for an integral assessment. Therefore, we developed new rules and proposed for each data type (FO, CV, FD and AC), weights for the different types of accuracy. The rules and their weights are based on expert judgement and were tested in WorldCereal.  In the Supporting information, Table S1 – step 6, we introduce the calculation of the confidence score. The protocol is explained in more detail in the document ReferenceDatasetsConfidenceScore Calculations.pdf available via DOI 10.5281/zenodo.7584463. | Manuscript, supporting information |
| **Reviewer 2** | | |
| 1) The WorldCereal reference database is strongly unbalanced in terms of geographical coverage (UE mainly). Even if it is a bit discussed, more discussion is needed on that point and particularly on what implication for global mapping (which is the main purpose of this database)? | The harmonized reference data set, described in this paper, served not only the WorldCereal project. The data can be used for all kind of mapping initiatives focussing on certain regions, crops and/or years. While for certain local applications the data set could be rather complete, this is not the case at global level. Global initiatives need to define approaches to deal with data gaps. Defining such approaches was not an objective of this paper. We refer to another WorldCereal paper (under review) to learn more about strategies to anticipate on the unequal distribution of reference data over years and space.  The discussion in the manuscript have been adapted to reflect this point.  Obviously, the harmonized data set described in this paper is just a first step. During our work we engaged with the GEOGLAM initiative (Group on Earth Observations Global Agricultural Monitoring Initiative), and we contributed to a new working group around in-situ data. In our paper we recommend an active role of GEOGLAM and the new working group encouraging the community to publish reference data and to help filling gaps in time and space and to achieve a more balanced distribution over crop types | Manuscript (line 294-295) |
| 2) In terms of information provided in the database, there is the land cover, crop type and irrigation status. I was wondering why information such as the agroecological systems or the type of farms (smallholder, medium, large, cf. Samberg 2016) or the field size (Fritz 2015) have not been included since it could help to refine crop mapping? | The aim of our harmonized reference data set is to provide training, test, and validation data for landcover and crop type mapping at the global level. This does not include other aspects of farming systems like farm type and field size, also because data sets, including these aspects, are sparse. We do agree that capturing extra aspects of farming systems could be of interest for downstream applications targeting different type of land use. In our discussion we have adapted our recommendation to include this point. | Manuscript (line 291-293) |
| 3) L143. Table 1. How the crops such as sugarcane or cassava that can have a crop cycle that can last up to 24 months have been considered? From your Table 1, annual cropland is for a period of 12 months but can we considered sugarcane or cassava as perennial crops? Not sure. | We followed the GEOGLAM-JECAM guidelines. The reference is given in the caption of table 1. The JECAM guidelines define annual cropland as follows: “The annual cropland from a remote sensing perspective is a piece of land of minimum 0.25 ha (min. width of 30 m) that is sowed/planted and harvestable at least once within the 12 months after the  sowing/planting date. The annual cropland produces an herbaceous cover and is sometimes combined with some tree or woody vegetation”. The guidelines indicate several exceptions. One exception concerns the sugarcane plantation and cassava crop which are  included in the cropland class although they have a longer vegetation cycle and are not yearly planted.  We added this exception to the description of annual crop land in table 1. | Manuscript (table 1) |
| 4) L150-151. How have you dealt with the case of cropping season spanning two calendar year? | In case only the year of observation is given, and the cropping season crosses the year calendar boundary, we consulted background information (paper, survey guidelines etc.) to map the label data to the correct calendar year. These decisions have been documented in an external document explaining the harmonisation and is available for each individual dataset that was harmonized.  We clarified the mapping between the year of observation and the calendar year. | Manuscript (line 153-154) |
| 5) L226. The Y-axe label of Figure 3 is a bit confused. A total number is not a percentage. | We updated the labels of the Y axis. | Manuscript (Figure-3-a and Figure-3-b) |
| 6) L269. Typing error at WorldCereal. | We corrected this typing error. | Manuscript (line 271) |
| 7) L270. In your table 2, there are several data-sets with year starting in 2016. Along your document you mention at many places that only the data from 2017 are collected. This is a bit confusing. | This is an inconsistency in our paper. Thanks for spotting this as we overlooked it. We did include 2016 in our data search and harmonization although the data from 2017 onwards is more useful in case downstream mapping initiatives would like to work with recent high resolution feature data (e.g. Sentinel 1 and 2).  We have changed the year 2017 into 2016 at several places in the manuscript. | Manuscript (different places) |
